# Infection kinetics and antibody responses in Deccani sheep during experimental infection and superinfection with bluetongue virus serotypes 4 and 16

**DOI:** 10.14202/vetworld.2019.41-47

**Published:** 2019-01-07

**Authors:** Kalyani Putty, Abdul Muzeer Shaik, Shaik Jahangeer Peera, Y. Narasimha Reddy, P. P. Rao, Sunil R. Patil, M. Shreekanth Reddy, B. Susmitha, J. Shiva Jyothi

**Affiliations:** 1 Department of Veterinary Microbiology and Veterinary Biotechnology, College of Veterinary Science, Rajendranagar, P V Narasimha Rao Telangana Veterinary University, Hyderabad, Telangana, India; 2 Veterinary Dispensary, Department of Animal Husbandry, Labbipet, Vijayawada, Andhra Pradesh, India; 3 Biovet, KIADB Industrial Area, Malur, Karnataka, India; 4 Ella Foundation, Genome Valley, Shamirpet, Hyderabad, Telangana, India

**Keywords:** bluetongue, bluetongue virus-16, bluetongue virus-4, infection kinetics, superinfection

## Abstract

**Aim:**

The current study was designed to understand the infection kinetics and antibody responses of major circulating serotypes of bluetongue virus (BTV) in India, i.e., BTV-4 and BTV-16 through experimental infection and superinfection of Deccani sheep, a popular breed of sheep found in the southern states of India.

**Materials and Methods:**

Experimental infection with 10^6^ TCID_50/ml_ BTV-4 was followed by superinfection with BTV-16 and vice versa. Along with observing for clinical signs and immunological responses in the experimentally infected sheep, the effect of infection of one specific serotype on the outcome of superinfection with a different serotype was also studied.

**Results:**

Certain interesting findings have been made in the course of experimental infection, such as prominent signs of infection in BTV-4 infection, mild or no clinical signs in BTV-16-infected and superinfected animals, and non-seroconversion of one of the BTV-16-superinfected animals. In addition, BTV was isolated from infected sheep in all the experimental conditions except BTV-16 superinfection. Furthermore, it was observed that immune response in the form of type-specific antibodies was slower with BTV-16 superinfection.

**Conclusion:**

Superinfection of a sheep with more than one serotype of BTV is a common phenomenon in BT endemic countries like India. Such situation was replicated in an experimental infection in the current study, and the findings to our knowledge are first of a kind and are likely to aid in unfolding the newer aspects of BTV pathogenesis and virulence.

## Introduction

Bluetongue (BT) is an infectious disease of wild and domestic ruminants, caused by BT virus (BTV), a double-stranded RNA virus of the genus *Orbivirus* within the family *Reoviridae* [1]. Twenty nine serotypes of BTV have been recognized to date [2,3], and a total of 24 serotypes have been reported from India [4,5]. A very flexible reassortment involving any of the genomic segments contributes majorly to the observed phenotypic variations in the BTV strains [6]. Although VP2 protein encoded by the highly variable segment 2 is the determinant of serotype, the other viral protein VP5 is also known to codetermine serotype along with VP2 [7]. Diagnosis of BT traditionally required isolation of virus and standard serological methods; however, recent advances in molecular biology made antigen and nucleic acid detection assays much simpler [2,8]. Curtailing the initial introduction into regions which harbor susceptible host and vector species and vaccination of the susceptible animal species may aid in effective prevention and control of BT [9]. The severity of the disease varies, not only between different species but also between different breeds within the same species. This can possibly be attributed to the basic differences in the inherent susceptibility of the cells of different hosts to BTV infection [10], the nature of the inflammatory mediators, and active components of the vascular system which take part in eliciting the response to infection [11,12]. The clinical manifestation of BT appears to be an outcome of the interplay of several factors in a fashion yet to be understood. Several studies including experimental infection of highly susceptible hosts such as sheep have essentially been carried out by different investigators worldwide to understand the nature of such interplay [13]. Such studies are helpful not only in unraveling the fundamental events in the pathogenesis of the disease but also in understanding the active role played by the host immune system and the design of effective control strategies. The history of experimental infections dates back to the late 18^th^ century, but those of more systematic nature were only reported in the early 20^th^ century. Scientists have tried different routes of inoculation of the virus [14,15]. Interestingly, blood from an infected sick sheep was found to be effective in establishing the infection in a susceptible host [16].

Studies on experimental infections have significantly contributed to our understanding of the pathogenicity, viral replication, virulence, induced immune response, transmission of the disease as well as reproductive failures due to BTV infection [17]. From these studies, it is very much apparent that, besides the host system, several aspects of the infecting virus including its serotype contribute to the great variability in the clinical outcome of the disease. The various serotypes of BTV, those that have been isolated to date, exhibit greater genetic heterogeneity which is also reflected in the degree and pattern of virulence [10]. This is an important aspect that needs to be studied to a greater extent.

To our knowledge, until now, no studies were reported on infection kinetics of Indian isolates of BTV-4 and BTV-16 with very few being reported on BTV-4 in other countries [18,19]. With this perspective, the present study was aimed to understand the pathogenesis of and immune responses to BTV serotypes 4 and 16 on their experimental infection and superinfection in Deccani breed of sheep.

## Materials and Methods

### Animals and ethical approval

The institutional animal ethics committee approval was obtained before beginning of the study. Sheep of Deccani breed was maintained in insect-proof facility at the sheep farm of Instructional Livestock Farming Complex, College of Veterinary Science, Hyderabad. The sheep used in the study were those which were confirmed to be BTV seronegative by c-ELISA. Eight such seronegative Deccani breed sheep between the ages of 6 months and 1 year were selected, of which three were inoculated with BTV-4 (09/NLR/15), three with BTV-16 (08ALG/15) isolated from field outbreaks of BT in Andhra Pradesh in 2014, and the remaining two animals were kept as control. This animal grouping was the same for both the models of infection and superinfection. Animals were provided with feed, fodder, and water *ad libitum* throughout the study for 2 months.

### Virus

The sheep were inoculated with 1 ml of plaque-purified BTV-4 and BTV-16. TCID_50/ml_ of virus isolates used for inoculation was 10^6^ for both BTV-4 and BTV-16. BTV-4 and BTV-16 adapted in BHK-21 cell line were cultured in *Culicoides sonorensis* (KC) cell line for 10 days at room temperature (without CO_2_ supplementation) before inoculating in sheep. Superinfection was done on 31^st^ day of primary infection (for both the serotypes 4 and 16). The inoculation was done intradermally in front of cranium of scapula at the shoulder region. The responses were monitored for 30 days after infection and superinfection.

### Post-inoculation follow-up and investigations

#### Clinical signs

The inoculated animals were examined daily for clinical changes in nasal, lingual, and oral mucous membranes, interdigital spaces, and coronary bands besides recording rectal temperature for 60 days in both the models.

#### Blood sampling and BTV detection

Blood samples were collected in either plain (for serum) or ethylenediaminetetraacetic acid-coated vacutainers (for virus isolation) (Becton Dickinson) of 5 ml capacity from animals inoculated with virus as well as control animals. For serum separation, vacutainers were centrifuged at 4000 rpm for 15 min at 4°C, and serum was separated in 1.5 ml microfuge tubes for c-ELISA and serum neutralization test (SNT). The serum collected was inactivated at 56°C for 30 min and stored at −20°C. For virus isolation and detection from infected animals, blood samples were processed as follows: 1 ml of blood from infected animals was taken into a 2-ml microfuge tube and was centrifuged at 1000 rpm for 10 min to remove plasma. The cell pellet was washed twice with 1×phosphate buffered saline (PBS), and to 500 μl of RBC pellet, 500 μl of sterile water was added, and the mixture was vortexed for 1 min. This was used for infection of KC cell culture. Virus isolation was done by one passage in KC cells (10 days) followed by three passages in BHK-21 cells (3 days each). Confirmation of cell cultures positive for BTV as seen by CPE characteristic of BTV was done by real-time polymerase chain reaction (PCR) with group-specific PCR by NS3-specific primers [20], followed by type-specific confirmation by BTV-4 and BTV-16-specific primers [2].

#### Immune response to BTV

Animals were tested for immune responses to BTV in the form of group-specific as well as serotype-specific antibodies.

#### c-ELISA

c-ELISA was done with BTV Antibody Test Kit, (cELISA, Veterinary Diagnostic Technology, Inc., U.S veterinary License #336) to screen the serum samples for antibodies to BTV following manufacturer’s protocol. The OD values were taken at 490 nm wavelength using microplate reader. The percentage inhibition (PI) of binding of the monoclonal antibody by a test serum was calculated as per kit’s instructions, and samples with a PI value <50% were considered negative, while those with a value >50% were considered as positive for BTV.

#### SNT

To detect serotype-specific antibodies, SNT was done in the 1^st^ month to detect antibodies specific to that serotype of the virus used for primary infection and in the 2^nd^ month to detect antibodies to both the serotypes used for infection and superinfection. Briefly, 1:10 serum dilution was prepared in maintenance medium in sterile deep well plate with the volume of 250 μl. 250 μl of medium containing 100 TCID_50/mL_ virus (either BTV-4 [09/NLR/15] or BTV-16 [08ALG/15]) per 50 μl was added to each well. The serum and virus mixtures were incubated for 1 h at 37°C with 5% CO_2_. 100 μl of incubated mixture was added in quadruplicates to each well of a 96-well microtiter plate that was seeded with 10^4^/100 μl of BHK-21 cells a day before. Four wells were kept as virus controls and four wells as cell controls in each plate. The plates were incubated for 5 days at 37°C with 5% CO_2_, and CPE readings were noted from 3 to 5 days. The plates were crystal violet stained on the 5^th^ day as follows: The medium from the plate was discarded and the wells were washed with 1×PBS (200 μl/well). The cells were then fixed in 10% formaldehyde (200 μl/well) at room temperature for 1 h, after which formaldehyde was discarded, and the plate was washed twice with 1×PBS. 1% crystal violet solution (200 μl/well) was added, and the plate was incubated for 1 h, after which the stain solution was discarded, and the wells were washed thrice with 1×PBS. The plates were air dried and observed for adherent monolayer as an indication of neutralization.

## Results

### Clinical signs of BT in the BTV-inoculated sheep

**Figure-1:**
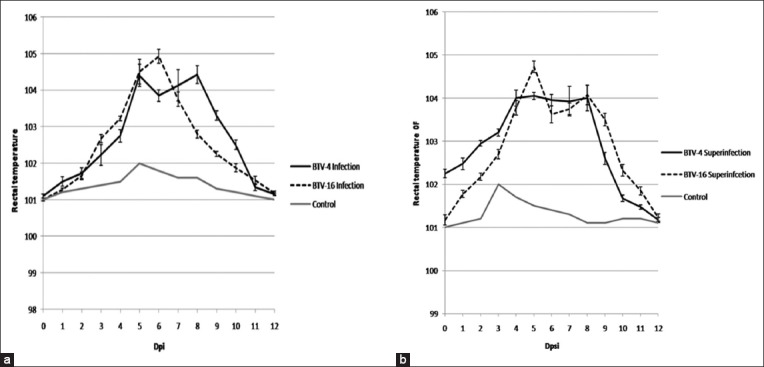
Clinical temperature profile of bluetongue virus (BTV) infection and superinfection. (a) Comparison of rectal temperature between animals infected with BTV-4 and those infected with BTV-16. (b) Comparison of rectal temperature between animals superinfected with BTV-4 and those superinfected with BTV-16. Maximum time taken to reach peak temperature for BTV-4-inoculated sheep was 8 days (irrespective of type of infection-primary or super). In case of BTV-16, peak temperatures were recorded as late as 6 days (primary infection) and 9 days (in case of superinfection). The graph is plotted by taking an average of temperature values of all the animals in each group on the respective days.

**Figure-2:**
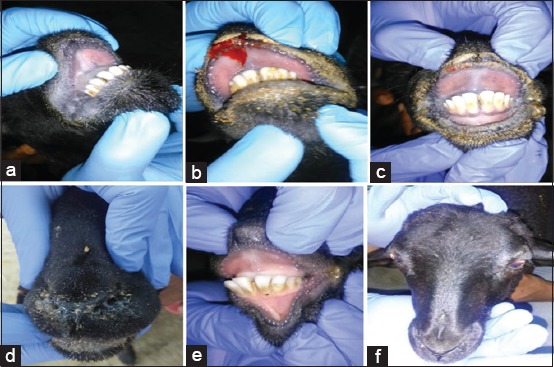
Clinical signs are observed in bluetongue virus (BTV)-4-infected animals but not in BTV-16 and either of the superinfection models. Clinical signs of BT including salivation (a) hyperemia of the lips (b) excoriation of the lips (c) and nasal discharges (d) were seen in BTV-4-infected animals. No clinical lesions were observed on infection with BTV-16 (e) Control animals exhibited no clinical signs throughout the duration of the study (f).

Rectal temperatures were recorded for all the BTV-inoculated as well as control animals every day for 60 days. Both BTV-4 and BTV-16 infected as well as superinfected animals showed a rise in the rectal temperatures, whereas the control animals exhibited no pyrexia. All the infected sheep showed a rise in rectal temperatures 5-8 days post-infection (dpi) in BTV-4 infection ranging from 103.7°F to 105.4°F and 4-8 days post-superinfection (dpsi) with BTV-16 ranging from 103.3°F to 105°F. In the model of BTV-16 infection, peak temperatures were recorded 5-6 dpi ranging 104.6°F-105.4ºF and 5-9 dpi in BTV-4 superinfection ranging 103.8ºF-104.9ºF (Figure-1). All the BTV-4-infected animals exhibited salivation 9 dpi (Figure-2a), two of the animals showed hyperemia of the upper and lower lips 8 dpi (Figure-2b), while one of these two animals also exhibited excoriation of epithelium on inner side of lips 9-11 dpi (Figure-2c). All the BTV-4-infected sheep exhibited mucous discharges from the nasal route (Figure-2d), while control animals exhibited no clinical signs (Figure-2f). Furthermore, no clinical lesions were observed on infection with BTV-16 (Figure-2e) except for pyrexia. Interestingly, neither of the animals superinfected with BTV-4 and BTV-16 had shown any clinical signs.

### Immunological responses to BTV

#### Group-specific antibody response

**Figure-3:**
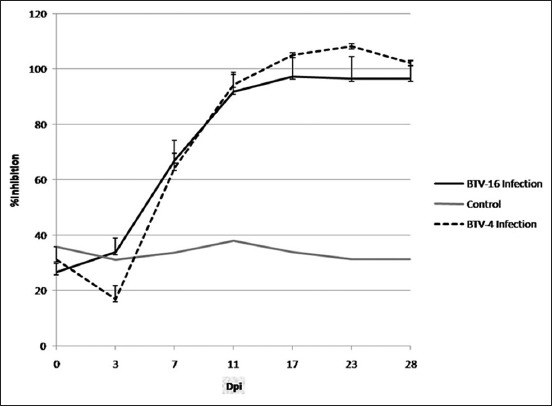
Group-specific antibody response to bluetongue virus (BTV)-4 and BTV-16 infection. By cELISA, group-specific antibodies to BTV-4 were detected by 5 dpi in one animal and 7 dpi in the remaining sheep, whereas in the BTV-16 infected, the same was detected by 7 dpi. The graph is plotted by taking an average of PI values of all the animals in each group on the respective days.

Group-specific antibodies to BTV were detected in the BTV-4-infected sheep by 5 dpi in one animal and by 7 dpi in the remaining sheep, whereas in the BTV-16 infected, the same was detected by 7 dpi (Figure-3). All the infected animals remained seropositive for BTV until the end of the study.

#### Type-specific neutralizing antibody response

SNT was done for serum samples up to 1 month from the day of primary infection and superinfection. It appeared that serotype-specific antibodies to BTV-4 can be detected 7 dpi as well as 7 dpsi. BTV-16-specific antibodies were detected in the serum of infected animals 7 days and 15 days after infection and superinfection, respectively. Furthermore, in one of the BTV-16-superinfected animals, seroconversion to BTV-16 was not observed until the end of the study (Table-1).

**Table-1:** Type-specific antibody profile during BTV-4 and BTV-16 infection and superinfection.

Dpi	BTV-4 infection				BTV-16 infection			
		
No.	Animal tag #207	Animal tag #208	Animal tag #231	Control	Animal tag #209	Animal tag #214	Animal tag #215	Control
7	+	+	+	−	+	+	+	−
11	+	+	+	−	+	+	+	−
17	+	+	+	−	+	+	+	−
21	+	+	+	−	+	+	+	−
28	+	+	+	−	+	+	+	−

**Dpsi**	**BTV-16 superinfection**				**BTV-4 superinfection**			

7	−	−	−	−	−	+	+	−
15	+	−	+	−	+	+	+	−
21	+	−	+	−	+	+	+	−
29	+	−	+	−	+	+	+	−

Dpi=Days post-infection; Dpsi=Days post-superinfection; (+)=Presence of neutralizing antibodies; (−)=Absence of neutralizing antibodies. Serotype-specific antibodies to BTV-4 appeared 7 days post-infection as well as superinfection (except in animal #209). BTV-16-specific antibodies were detected in the serum of infected animals 7 days and 15 days after infection and superinfection, respectively. Furthermore, in one of the BTV-16-superinfected animals (#208), seroconversion to BTV-16 was not observed until the end of the study. BTV=Bluetongue virus

#### BTV isolation from infected blood samples

**Figure-4:**
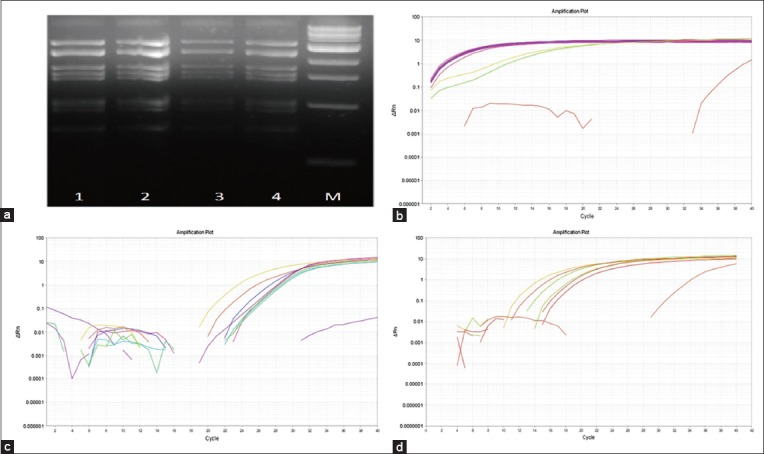
Isolation and confirmation of bluetongue virus (BTV) from experimentally infected sheep. (a) Agarose gel electrophoresis of RNA extracted from CPE exhibiting BHK-21 cell culture supernatants infected with blood of infected and superinfected animals collected on days of peak pyrexia shows typical segmented pattern of BTV dsRNA. Lanes 1 and 2 show RNA pattern of BTV isolated from animal #207 and #208 on BTV-4 infection. Lanes 3 and 4 show RNA pattern of BTV isolated from animal #209 on BTV-16 infection and BTV-4 superinfection, respectively. Similar pattern was observed for all the other BTV-4-infected and superinfected and BTV-16-infected animals. RNA could not be isolated from BTV-16-superinfected animals. (b) Confirmation of BTV by NS3 group-specific polymerase chain reaction (PCR). (c) Confirmation of BTV-4 by type-specific PCR. (d) Confirmation of BTV-16 by type-specific PCR.

Blood collected from infected and superinfected animals on days that recorded highest rectal temperatures, i.e., 5-8 dpi (BTV-4), 5-6 dpi (BTV-16), 4-8 dpsi (BTV-4), and 5-6 dpsi (BTV-16) was processed for virus isolation in KC cells followed by three passages in BHK-21 cell lines. CPE characteristic of BTV in BHK-21 cell cultures was observed with both BTV-4- and BTV-16-infected and BTV-4-superinfected animals but not in BTV-16-superinfected animals and in uninfected control animals. RNA extracted from CPE exhibiting cell culture supernatants exhibited a characteristic segmented pattern of BTV double-stranded RNA on agarose gel (Figure-4a). Real-time PCR analysis confirmed the virus isolated to be BTV by group-specific NS3 PCR (Figure-4b). Corroborating CPE findings, neither BTV RNA could be isolated from any of the BTV-16-superinfected animals, nor they were tested to be positive for group-specific BTV confirmation by NS3-specific PCR. Type-specific real-time PCR analysis confirmed isolation of BTV-4 and BTV-16 in BTV-4- and BTV-16-infected and superinfected animals (only BTV-4), respectively (Figure-4c and d).

## Discussion

Early detection of BT in the affected population is crucial, for which an understanding of the clinical signs is essential. However, depending on the species and breed of the animal affected and serotype of the virus, certain cases of infection go unnoticed until a fulminant infection is established. Even more complicated are superinfections with different serotypes. Thus, it is essential to understand the infection kinetics of major circulating serotypes of BTV. Experimental infection of most commonly affected animal population might be of help in this regard. The present study was carried out with an objective to understand the infection kinetics and immunological effects elicited by the infection of BTV serotypes 4 and 16. We have also looked at the consequences of superinfection with these serotypes. Thus, we have created two different models of experimental infection; one in which the sheep were infected first with BTV-4 followed by BTV-16 and the other one infected first with BTV-16 followed by BTV-4 and compared the changes thereof. The findings from this kind of a study are essential as they might help in getting a clear understanding of events that actually take place in the clinical cases of these most commonly observed superinfections in BT endemic areas. BTV-4 and BTV-16 were considered for this type of a study keeping in view of the high prevalence of infections and superinfections with these serotypes in the southern states of India (data not published yet) as well as the observed less relatedness of both the serotypes to each other [21].

In the current study, infection with BTV-4 had resulted in the appearance of prominent clinical signs similar to those observed in the experimental infection with BTV-4 done previously [19]. The endothelial injury caused by the virus due to its replication besides the inflammatory and vasoactive mediators secreted in response to the viral infection possibly explains the observed clinical signs including lesions associated with the infection. With BTV-16 infection (followed further by BTV-4 superinfection) and superinfection with BTV-16 of BTV-4-infected animals, no such clinical signs were observed. This was also the case reported by other investigators when they tried to infect susceptible species of animals with field strains from cases of fulminant infection [12,19,22]. Another study that involved BTV-16 serotype for vaccination also marked no clinical reactions [23]. Several aspects of the virus, as well as host, contribute to such variations in the outcome of the disease making BT known for its highly variable clinical spectrum [24,25]. Among those factors, propagation of certain serotypes of BTV in cell cultures is one and was found to negatively influence the virulence [12,19,22]. KC cell adaptation before inoculation of virus in experimental animals was, hence, implemented in this study to mimic the field scenario to the maximum extent possible.

Furthermore, as reported by some researchers, the titer of the virus preparation used for inoculation could also possibly determine the virulence of that serotype used for infection [23,26]. Thus, the viral titer 10^6^ TCID_50/ml_ of BTV-4 might have had sufficient virulence to produce considerable clinical signs, whereas the same titer might not have been enough for BTV-16. However, both the groups of animals showed a rise in rectal temperatures. In addition, a considerable alteration of hematological parameters was recorded in the current study in both the models of infection, namely increased TLC and decreased TEC and hemoglobin content (data not shown), a feature typical of viral infections.

Interestingly, BTV-16 could not be isolated from any of the superinfected sheep in spite of development of BTV-16-specific neutralizing antibodies although much later (15 dpsi) than its counterpart superinfection with BTV-4 (7dpsi). There were reported cases of even natural infections, where BTV could not be isolated through propagation in cell cultures and that such kind of blood was not infectious to vector insects [10,27]. Although cross-neutralization of BTV-16 with BTV-4-specific neutralizing antibodies circulating in the superinfected animals is highly unlikely given that both the tested serotypes are serologically distant [21], it is possible that BTV-16 isolate used in the current study might not be a highly pathogenic virus. Substantiating this statement is the finding that both BTV-16-infected and superinfected sheep in the current study showed less apparent clinical signs. Considerable variations in the time of onset of group- and type-specific antibodies for the two tested serotypes were a major observation in the current study. In one of the BTV-16-superinfected animals, seroconversion did not happen until the end of the study which was also observed by other researchers wherein, in experimental infection of Dorset Poll sheep with BTV-26, one of the animals did not seroconvert until the end of the study [28]. Thus, it is possible that, within the same flock, pathogenesis, disease severity, and clinical signs may vary among the animals [24,25]. Neutralizing antibodies specific to BTV-4 serotype appeared quickly irrespective of the type of infection. Detection of neutralizing antibodies 15 dpsi for BTV-16 probably reflects a delayed immune response to superinfection with BTV-16. Similar kind of findings was reported by other researchers where BTV-1 superinfection in calves that were experimentally infected with BTV-8 showed low titers of neutralizing antibodies to BTV-1 by neutralization assay [29].

Although in the current study, titration of antibodies was not done, the time taken for onset of neutralizing antibodies in BTV-16 superinfection was 15 days when compared to 7 days in BTV-4 infection and superinfection. As mentioned before, cross-neutralization of BTV-16 with BTV-4-specific neutralizing antibodies circulating in the superinfected animals is highly unlikely in this scenario as cross-protection conferred by BTV-4 which was experimentally confirmed for only BTV-9 and BTV-11 [30]. Furthermore, parallel studies in our laboratory suggested cross-neutralization of BTV-10 and not BTV-16 with BTV-4 serum. On the other hand, reports on BTV-16 cross-neutralization studies are not reported to our knowledge, until now. Studies on role of cell-mediated immune responses in BTV-4 and BTV-16 infection can shed better light into understanding findings as reported here. The current study raises several important questions that need to be answered concerning the differences observed between the two serotypes in the clinical outcome and immunological responses to the virus. Nevertheless, these kinds of observations may aid in taking our understanding of the pathogenicity of different BTV serotypes, their relatedness to each other, and the phenomenon of cross-protection, to the next level. They also stress the need to carry out studies in this direction to clarify and/or confirm these interesting findings, which can be of help in designing strategies to formulate vaccines that are effective in conferring immunity to a broad spectrum of BTV serotypes.

## Conclusion

Under experimental infection, the infection kinetics of BTV-4 and BTV-16 are characteristic to each serotype and vary significantly from each other. BTV-4 appeared to delay the immune response to BTV-16 and thus may partially cross protect but not to the extent where it completely curtails the infection, which needs to be further characterized by future investigations.

## Authors’ Contributions

KP, YNR, and PPR designed the study; KP, AMS, SJP, SRP, MSR, BS, and SJJ performed the experiments; KP, YNR, and JSJ analyzed the data and wrote the manuscript. All authors read and approved the manuscript.
